# Catalytic Pyrolysis of Polypropylene for Cable Semiconductive Buffer Layers

**DOI:** 10.3390/polym16101435

**Published:** 2024-05-19

**Authors:** Xiaokai Meng, Hua Yu, Zhumao Lu, Tao Jin

**Affiliations:** State Grid Shanxi Electric Power Research Institute, Taiyuan 030001, China; yuhua@163.com (H.Y.); luzhumao@163.com (Z.L.); jintao@163.com (T.J.)

**Keywords:** cable, buffer layer, polypropylene, pyrolysis, principal component analysis

## Abstract

With the progress of the power grid system, the coverage area of cables is widening, and the problem of cable faults is gradually coming to affect people’s daily lives. While the vast majority of cable faults are caused by the ablation of the cable buffer layer, polypropylene (PP), as a common cable buffer material, has pyrolysis properties that critically impact cable faults. Studying the semiconductive buffer layer of polypropylene (PP) and its pyrolysis properties allows us to obtain a clearer picture of the pyrolysis products formed during PP ablation. This understanding aids in the accurate diagnosis of cable faults and the identification of ablation events. In this study, the effects of temperature and catalyst (H-Zeolite Standard Oil Corporation Of New York (Socony) Mobil-Five (HZSM-5)) content on the PP thermolysis product distribution were studied by using an online tubular pyrolysis furnace-mass spectrometry (MS) experimental platform. The results showed that PP/40% HZSM-5 presented the highest thermolytic efficiency and relative yield of the main products at 400 °C.

## 1. Introduction

In recent decades, with the rapid development of the economy and society, the cable coverage area has been widening, which has promoted the development of the Chinese power system. However, over time, cable failure has gradually become a problem that cannot be ignored. In recent years, buffer layer ablation faults have occurred one after another in cross-linked polyethylene (XLPE)-insulated, corrugated-aluminum-sheathed cables of 110 kV and above in many locations in China, and similar faults have also occurred in Australia and other countries [1]. Buffer layer ablation is specifically manifested as a buffer layer and aluminum sheath inner wall with white spots and burn traces, a buffer layer and insulation shielding layer with discharge traces, and in severe cases, a buffer layer with obvious ablation holes. The semiconductive buffer layer is located between the metallic aluminum sheath and the cable insulation shield and consists of the winding tape and air gap. The main component of the wrap-around tape is polypropylene (PP), and in order to achieve semiconductive properties, the production process will be doped with carbon black, with a longitudinal water-blocking function thanks to the combination of the wrap-around tape also including water-absorbing resins [2]. These problems are mainly caused by semiconductive buffer layer ablation due to cable failures, seriously affecting the safe operation of the cable.

Due to the structural requirements and production process, the metallic aluminum sheath is closed. Therefore, when breakdown of the cable body insulation occurs, such as partial discharge, localized high temperature, cracking, etc., some characteristic gas products will be produced, and the nature, severity and trend of the defects can be judged through the analysis of these gas products [3]. Densley [4] found that partial discharges (PDs) lead to decomposition of cable insulation, which is mainly decomposed into gases. Arc faults produce large amounts of explosive gas by-products that lead to ablative holes in the insulation [5]. When low-voltage (LV) cables with aluminum conductors are fully submerged and the conductors are exposed, leakage of current to the surrounding or other conductors can lead to corrosion of the aluminum and release of hydrogen gas [6]. In addition, Mo et al. [7] used thermogravimetric and differential scanning calorimetry techniques to demonstrate that gases are generated during the pyrolysis of cross-linked polyethylene. Different cable failures (low- and high-temperature pyrolysis of semiconductors and insulating and buffer materials; low- and high-energy discharge activities of insulating materials and electrochemical corrosion of buffer materials) show different gas generation characteristics [8]. Detecting the characteristic gases released during the ablation of cable buffer layers is a new detection technique worth studying, but there is still a gap in the research on the application of gas detection to identify the ablation defects of buffer layers, and it is difficult to grasp the correlation between the changes in the properties of buffer layers in the process of ablation and the gas products.

In addition, there are different views on how to deal with the polymer plastic after metal recycling in the power system [9,10]. Regarding cable plastics, besides XLPE and PP, there are mainly polyvinyl chloride (PVC) and halogen-free flame-retardant polymer (HFFR). At present, most of the plastic in cables is incinerated and disposed of by landfill, but these methods have been gradually banned in recent years because of their environmental impact.

With the development of technology, the calorific value of waste plastics is higher than that of coal, and the specific gravity of oil is slightly lower. As such, it is a good fuel. If it is utilized, it cannot only solve the long-term environmental problems that waste plastics are difficult to deal with but also alleviate the energy problem appropriately, which will produce great economic benefits. Thermochemical conversion is widely used, with its main conversion forms the incineration method, pyrolysis method, and gasification method. Waste plastics can be cracked into hydrocarbons under high-temperature conditions and then processed to obtain fuel oil, coke, and other fuels. However, the main disadvantages of pyrolysis are the wide distribution of products and the need for a high temperature, and it is necessary to use catalysts to solve these problems [11]. Effective catalysts can control the distribution of products, improve their yield, and reduce the temperature required for their reaction. Among all the catalysts used in polymer pyrolysis, zeolite is considered to be the most effective because of its strong acidity and stereoscopic effect. A large number of studies have shown that among zeolites with different structures and pore sizes, H-Zeolite Standard Oil Corporation Of New York (Socony) Mobil-Five (HZSM-5) is the most suitable zeolite catalyst for the pyrolysis of plastics into hydrocarbons and gives the highest aromatic yield [12,13,14].

In addition, Saha et al. [15] studied the catalytic decomposition behavior of PP on HZSM-5 catalyst and showed that the presence of catalyst significantly reduced the maximum decomposition temperature T_m_ of PP samples, with an optimal catalyst percentage of about 50 wt% and a decrease in T_m_ of about 161 °C. Santos [16] et al., through experiments, showed that zeolite Y (USY) and zeolite HZSM-5 can be used as catalysts for the pyrolysis of municipal plastic waste and to reduce its degradation temperature. When HZSM-5 was used as catalyst, an increase in the solid fraction and a decrease in the liquid fraction were observed at 450 °C and 30 min.

Mass spectrometry has the advantages of high sensitivity and accuracy, rapid detection, etc. It can provide accurate molecular weight and structural information on samples at the same time, and it can be used in combination with other technologies, meaning is suitable for the analysis of complex systems [17,18]. For example, in a study of the application of the PP pyrolysis mass spectrometry technique and mass spectrometry coupled with a tubular pyrolysis furnace, where PP products were imported into the mass spectrum of the pyrolysis furnace through a transport channel, advantageous in situ detection was realized of the main product and intermediate and real-time online monitoring of the active material, which helped to build a comprehensive understanding of the pyrolysis product distribution and the PP pyrolysis model [19].

To sum up, the discharge ablation mechanism and gas production process of cable buffer strips have not been thoroughly studied, and the utilization of a polymer in cables should also be considered. Therefore, the pyrolysis mechanism of PP was summarized through real-time online analysis of the product distributions of samples under different pyrolysis temperatures and HZSM-5 catalyst ratios by using a tubular furnace-MS pyrolysis experimental platform. The comprehensive experimental results and theoretical analysis provide new research ideas for the ablation mechanism of the buffer layer [20], as well as a basis and reference for the improvement of the PP pyrolysis method.

## 2. Experimental Section

### 2.1. Materials

Basic information on the raw materials used in this study is listed in Table 1. Before the experiments, the catalyst was heated to 550 °C in a tubular furnace (model: OTF-1200X, from Hefei Kocrystal Materials Technology Co., Ltd., Hefei, China) with nitrogen at a flow rate of 200 standard cubic centimeters (SCCM) per minute for 5 h, and the temperature was measured by a K-type thermocouple (WRNK-191, from Yanli Automation Instrument Co., Ltd., Shanghai, China). In addition, a PX224ZH analytical balance (Changzhou Ohaus Company, Changzhou, China) with a measurement accuracy of 0.0001 g was used to ensure that the sample mass used for each measurement was precisely 40 mg. These samples were then thoroughly mixed with the catalyst. To explore the impact of catalyst concentration on pyrolysis performance, the catalyst loadings were systematically adjusted to 20%, 30%, 40%, and 50% by weight (wt%).

### 2.2. Device Setup

**Figure 1:**
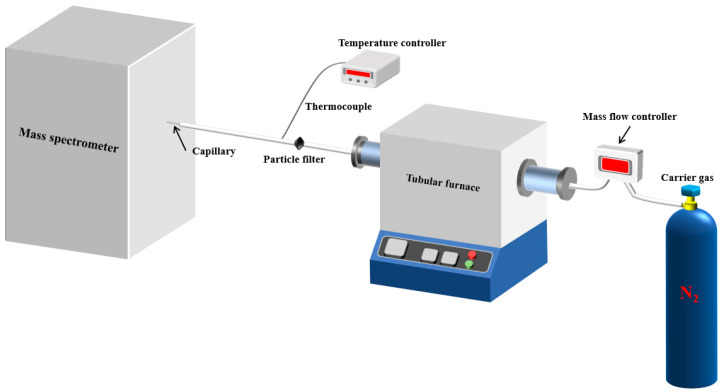
Schematic diagram of the pyrolytic single-photon ionization (SPI)-TOF-MS system.

As shown in Figure 1, the experimental setup consisted of a tubular furnace, a transfer tube for introducing samples, and a matrix-assisted laser desorption ionization (MALDI) time-of-flight (TOF) mass spectrometer (MS) (type: ultrafleXtreme, from Bruker Corporation, Billerica, MA, USA).

Samples were placed in a quartz boat and introduced into the tube furnace preheated to temperatures of 300 °C, 400 °C, 500 °C, and 600 °C. The heating process occurred in a nitrogen atmosphere. Once heated, the sample passed through a glass fiber filter and then a capillary tube before being introduced into the mass spectrometer for analysis.

In addition, the morphology of the catalyst was obtained by scanning electron microscopy (SEM) with a JSM-7001F microscope (JEOL Ltd., Tokyo, Japan). Furthermore, energy-dispersive spectroscopy (EDS) of the catalyst HZSM-5 was also conducted.

## 3. Results and Discussion

### 3.1. Morphology of Catalyst

**Figure 2:**
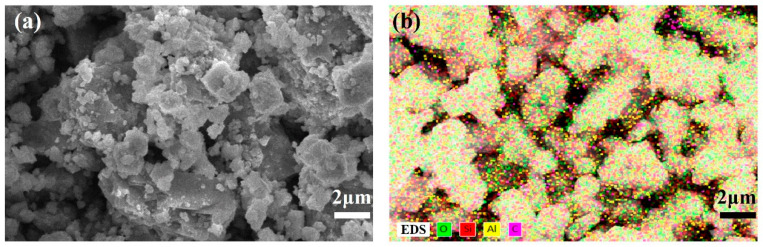
(**a**) SEM image and (**b**) EDS with elements O, Si, Al, and C of HZSM-5.

As shown in Figure 2, SEM measurement was applied for the research on the catalyst, with the size of HZSM-5 about 3 μm. In general, the interaction between polypropylene (PP) and the catalyst is characterized by surface contact. The relatively small particle size of HZSM-5 allows for effective interaction with PP. Given that most of the powder particles exceed 0.1 μm in size, the surface effect can be considered negligible. Furthermore, according to the elemental analysis from energy spectra the of catalyst, the main elements in HZSM-5 are O, Si, Al, and C, and a content of Si as well as Al was confirmed.

### 3.2. Mass Spectra of Volatile Products from Polypropylene Pyrolysis

**Figure 3:**
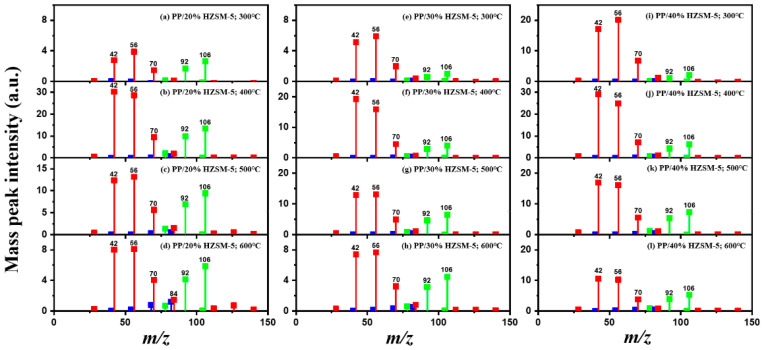
PP/20% HZSM-5, PP/30% HZSM-5, and PP/40% HZSM-5 of volatile pyrolysis products under different temperatures of mass spectrum: (**a**,**e**,**i**) 300 °C, (**b**,**f**,**j**) 400 °C, (**c**,**g**) 500 °C, and (**d**,**h**,**k**,**l**) 600 °C (the red line represents olefins, the blue line represents diolefins, and the green line represents aromatic hydrocarbons).

In order to explore the catalytic efficiency of the HZSM-5 catalyst, experiments with different catalyst ratios (20–40 wt%) were carried out at different reaction temperatures (300–600 °C), and the mass spectra of different volatile pyrolysis products at different temperatures were obtained, as shown in Figure 3. Different contents of HZSM-5 changed the distribution of pyrolysis products. For the mass spectra, all the pyrolysis products are hydrocarbons. Consequently, the molecular formulas of these compounds can be ascertained directly through photoionization mass spectrometry (PIMS). By analyzing the mass-to-charge ratio (*m*/*z*), the molecular formulas of the products can be accurately determined. Based on previous summaries, the various components could be easily distinguished, as shown in Table 2. The products are mainly divided into three categories: olefins, diolefins, and aromatic hydrocarbons. Among the aromatic hydrocarbons, the pyrolysis products are mainly benzene (*m*/*z* = 78), toluene (*m*/*z* = 92), and xylene (*m*/*z* = 106), which are also known as BTX. Except ethylene (*m*/*z* = 28), all products are generated through a series of reactions such as bond splitting, intramolecular hydrogen transfer, and β-fracture [21,22], while diene products are usually formed in secondary reactions [23], and aromatic compounds observed during pyrolysis are formed through the Diels–Alder reaction [24,25].

As shown in Figure 3a–d, the main products include propylene (*m*/*z* = 42), butene (*m*/*z* = 56), pentene (*m*/*z* = 70), toluene (*m*/*z* = 92), and xylene (*m*/*z* = 106). Their relative peak intensity first increases and then decreases with the increase in pyrolysis temperature, and it reaches a peak at 400 °C. The diene products have almost no relative intensity. Similarly, in Figure 3e–l, the content of catalyst is significantly increased, and for the main products, such as propylene (*m*/*z* = 42), butene (*m*/*z* = 56), pentene (*m*/*z* = 70), toluene (*m*/*z* = 92), and xylene (*m*/*z* = 106), their relative strength first increases and then decreases with the increase in pyrolysis temperature. It reaches its peak at 400 °C, and the diene products can hardly match its relative strength. These phenomena indicate that the HZSM-5 catalyst is selective in product formation, and that almost no secondary reactions occur during pyrolysis. In addition, with the change in catalyst content, the main pyrolysis products are always propylene (*m*/*z* = 42), butene (*m*/*z* = 56), and pentene (*m*/*z* = 70) with a higher relative strength, which may be related to the higher conversion rate of HZSM-5 catalyst and the selectivity of promoting PP degradation to light olefin. Similarly, due to the structural characteristics of HZSM-5 catalyst, benzene, toluene, and xylene (BTX) can be formed at a low temperature of 300 °C, indicating that the catalyst can significantly accelerate the formation of BTX.

Without considering the influence of the catalyst ratio, the relative intensity of pyrolysis products (the types and proportions of chemicals present in the pyrolysis products) at 300–400 °C always increases, but then it shows a downward trend, which may be related to the destruction of effective acid sites inside the catalyst by coke on the surface of zeolite and high temperature, resulting in the deactivation of the catalyst. For the HZSM-5 catalyst, the presence of stronger acid sites means that an increase in reaction temperature not only promotes polymer cracking but also leads to the conversion of lighter hydrocarbons into heavier hydrocarbons through continuous oligomerization, cyclization, and aromatization reactions, as shown in Figure 3a,b,e,f,i,j. As shown in Figure 3c,d,g,h,k,l, the relative abundance of the major pyrolysis products exhibits a downward trend between 500 and 600 °C. This trend is attributed to the formation of coke from the large volume of hydrocarbons on the catalyst’s surface at elevated temperatures. The coke is deposited within the pore structure of the zeolite, leading to pore blockage and consequently reducing the catalyst’s activity.

As shown in Table 2, 16 kinds of hydrocarbons with molecular weights of less than 150 were detected during the pyrolysis process. With the progress of the experiment, we found that the olefin and BTX were dominant among the pyrolysis products, while the formation of diolefin products was the minority. The presence of the HZSM-5 catalyst did not significantly improve the yield of diolefin products, indicating that the catalyst did not tend to produce diene products during the pyrolysis process.

In fact, the mechanism of pyrolysis is to produce free radicals, and typical reactions for these substances are β-lysis, isomerization, oligomerization, and Diels–Alder reaction to produce BTX and olefin [26,27,28,29,30]. The HZSM-5 catalyst has a high site density and strong acid site, which is conducive to the pyrolysis reaction of PP. However, its porous structure is an interconnecting hole with elliptical pores. This type of structure means that almost all the heavier hydrocarbons in the pyrolysis process of PP are difficult to diffuse and gradually accumulate to form coke blocking holes, thus reducing the activity of the catalyst. Therefore, in Figure 3, between 400 °C and 600 °C, we can find that the relative strength of the product decreases.

In summary, when the reaction temperature and catalyst ratio are optimized, the PP/40% HZSM-5 combination has the highest relative strength, which meets the requirements of obtaining low-quality olefin and BTX products.

### 3.3. The Intensity of the Main Pyrolysis Products versus Time

**Figure 4:**
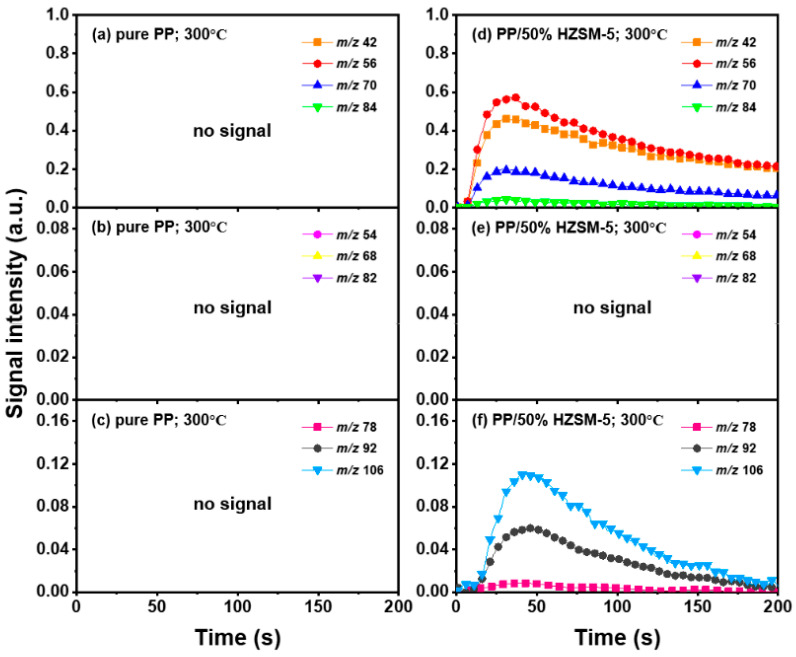
Peak evolution versus time curves of molecular ions from main products at 300 °C as (**a**–**c**): pure PP; (**d**–**f**): PE/50% HZSM-5.

**Figure 5:**
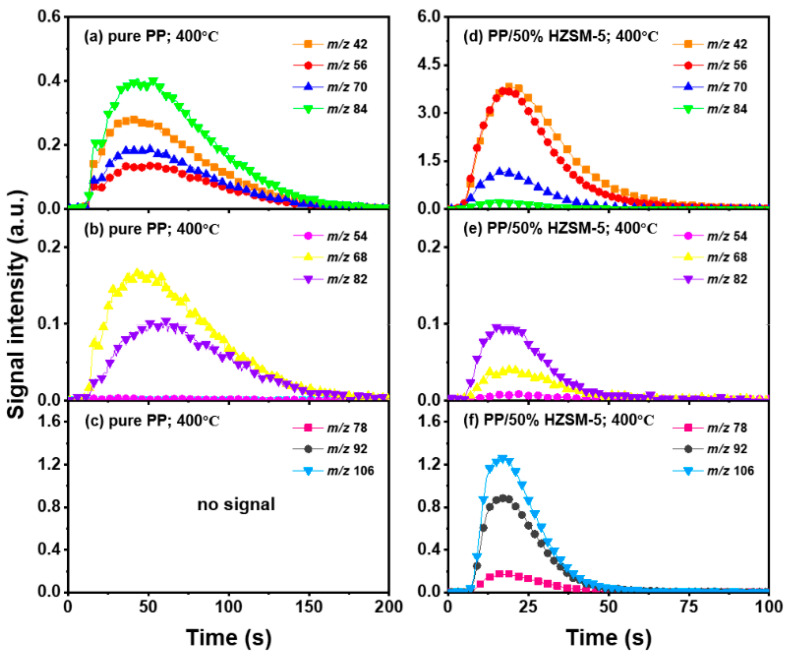
Peak evolution versus time curves of molecular ions from main products at 400 °C as (**a**–**c**): pure PP; (**d**–**f**): PE/50% HZSM-5.

Thanks to the excellent online detection capability of the SPI-TOF-MS platform, we can observe the time evolution curves of various products in real-time during the pyrolysis process. According to the analysis of Section 3.2, 300–400 °C is favorable to the pyrolysis process of PP, so we mainly analyzed the role of the HZSM-5 catalyst. We chose pure PP and PP/50% HZSM-5 for the experiment, as shown in Figure 4 and Figure 5. In Figure 4, at 300 °C, no signal could be detected for pure PP, indicating that PP could rarely be pyrolyzed at a relatively low temperature. Meanwhile, with the addition of zeolite, the production processing of the main alkene products propylene (*m*/*z* = 42), butene (*m*/*z* = 56), and pentene (*m*/*z* = 70) exceeded 200 s, while that of BTX ended in 200 s. However, there was still no signal of dienes for PP with the catalyst HZSM-5 at 300 °C. In Figure 5, at 400 °C, the signal of alkenes and dienes appears for pure PP pyrolysis, increasing obviously for PP with the catalyst, revealing that 400 °C was a more suitable pyrolysis temperature compared to 300 °C. Furthermore, the addition of HZSM-5 catalyst significantly reduced the thermal degradation temperature of PP and significantly increased the olefin yield, and it was selective to the product.

**Figure 6:**
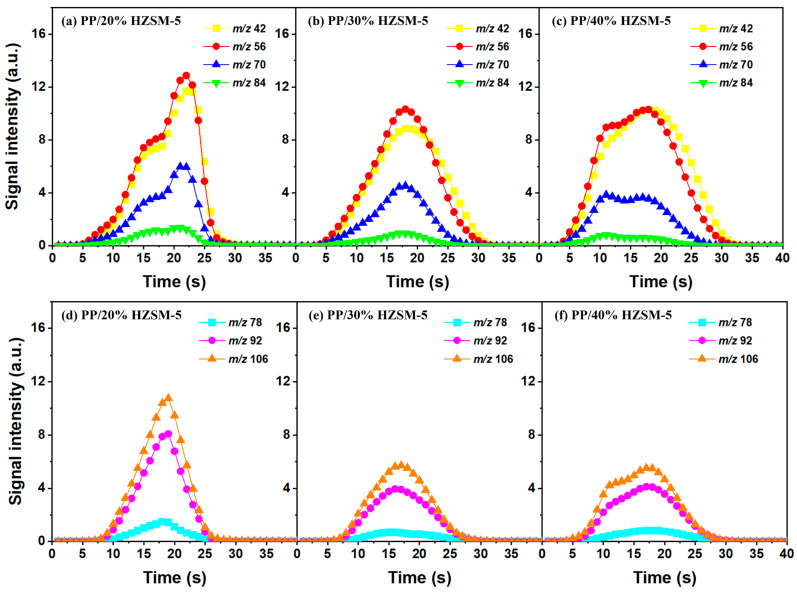
Time evolution curves of main pyrolysis products of PP with different HZSM-5 contents at 500 °C: (**a**,**d**) PP/20% HZSM-5, (**b**,**e**) PP/30% HZSM-5, (**c**,**f**) PP/40% HZSM-5.

Regarding olefin, in Figure 5a,d, the order of relative strength of pure PP pyrolysis products from high to low is 2-methyl-1-pentene/4-methyl-1-pentene (*m*/*z* = 84) > propylene (*m*/*z* = 42) > pentene (*m*/*z* = 70) > butene (*m*/*z* = 56). However, due to the presence of catalyst, the order changes to propylene (*m*/*z* = 42) > butene (*m*/*z* = 56) > pentene (*m*/*z* = 70) > 2-methyl-1-pentene/4-methyl-1-pentene (*m*/*z* = 84), indicating that the HZSM-5 catalyst is selective to pyrolysis products and has a tendency to transform to low-molecular-weight olefin. In addition, as shown in Figure 4d and Figure 5d, the relative strength relationship between propylene (*m*/*z* = 42) and butene (*m*/*z* = 56) changes slightly with the increase in temperature without considering the influence of the catalyst, and it tends to develop in the direction of propene generation. The relative strength of propylene and butene is always stronger than that of pentene (*m*/*z* = 70) and hexene (*m*/*z* = 84), indicating that the pyrolysis products of PP tend to produce alkenes within C4, and that they mainly tend to produce propylene and butene. Compared with Figure 5b,e, there is no formation of diene products in Figure 4b,e, indicating that a low temperature is not conducive to the formation of diene products. However, although diene products are generated, as shown in Figure 6, their relative strength is low and can be ignored. With the addition of the HZSM-5 catalyst, the relative strength of diene products decreases, indicating that the catalyst has the ability to inhibit the formation of diene products. When comparing Figure 4c,f and Figure 5c,f, it is obvious that with pure PP, it is difficult to generate BTX by pyrolysis, and that the addition of the catalyst significantly improves the relative strength of BTX; moreover, with the increase in temperature, the relative strength increases by several times and the reaction period is significantly shortened.

**Figure 7:**
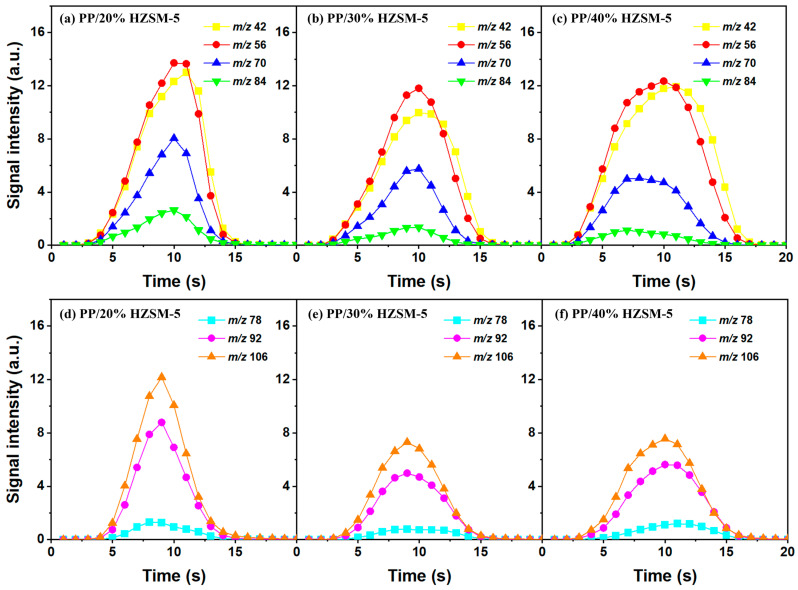
Time evolution curves of main pyrolysis products of PP with different HZSM-5 contents at 600 °C: (**a**,**d**) PP/20% HZSM-5, (**b**,**e**) PP/30% HZSM-5, (**c**,**f**) PP/40% HZSM-5.

Similarly, based on the analysis of Section 3.2, we chose to explore the influence of different catalyst ratios (20 wt%, 30 wt%, and 40 wt%) on the pyrolysis of PP; we did so due to the fact that almost no diene products were generated during the pyrolysis process and given the reduction in catalyst activity at 500–600 °C. Compared with Figure 7, the pyrolysis reaction time in Figure 6 is almost twice as long, indicating that a high temperature is conducive to reducing the pyrolysis reaction time. In addition, the relative strength of the products does not decrease significantly with the increase in the catalyst ratio, which is mainly due to the deactivation of the catalyst. There are two types of product curves (unimodal and bimodal), which indicates that different reaction mechanisms are involved in the catalytic pyrolysis of PP. HZSM-5 zeolite is considered to be one of the most effective catalysts for polymer catalytic thermal degradation due to its strong acidity, high acid site density, and small pore size. However, the presence of a small pore size hinders the entry of large molecular chains into the acidic sites located in the cavity, which gradually accumulates and blocks the pores, resulting in the deactivation of the catalyst. However, the relative intensity of BTX does not significantly decrease, indicating the existence of another pyrolysis mechanism that produces BTX.

**Figure 8:**
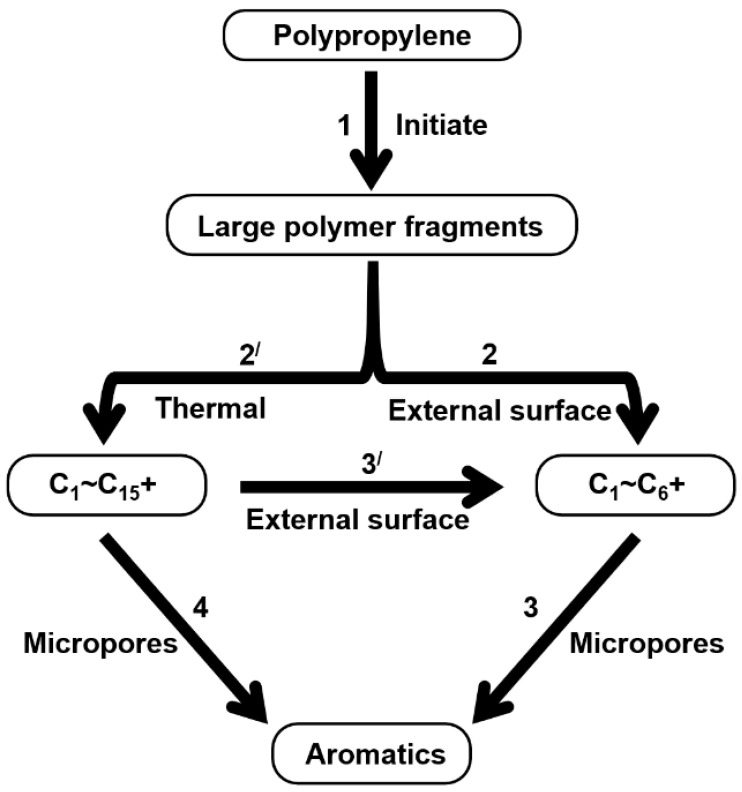
Schematic diagram of thermal degradation mechanism of PP/catalyst.

Therefore, based on the previous analysis, two decomposition stages are indicated. At the beginning of decomposition, large polymer fragments decompose on the outer surface of HZSM-5 zeolite, forming small molecular chains and free radicals. The same reaction occurs during non-catalytic degradation. Thus, there are two paths (as shown in Figure 8), one for catalytic degradation and the other for non-catalytic degradation. Shape selection reactions take place in internal channels of zeolite, where further pyrolysis and oligomerization of molecular chains lead to the generation of olefin and aromatic hydrocarbons. There is no shape selectivity on the outer surface of zeolite due to there being no space limitation. Large molecular chains and a small amount of active light hydrocarbons are converted at the acid sites outside the catalyst in two main ways: (a) continued cracking into smaller olefins; (b) a polymerization reaction occurs to form BTX [31,32,33]. Therefore, there is a delay in the reaction between the olefin produced on the outer surface and BTX, which shows as two peaks in the product diagram, as illustrated in Figure 7a,c,f. Later, with the increase in reaction temperature, the reaction rate accelerates, which is not obvious in the diagram of pyrolysis products.

In addition, the performance of the catalysts with different mixing ratios shows the same trend. A lower reaction temperature limits the reaction rate, but a too-high temperature will lead to the deactivation of the catalyst. Therefore, for the pyrolysis experiment of PP, it is necessary to find an appropriate reaction condition, that is, the PP/40% HZSM-5 combination sample at 400 °C.

## 4. Conclusions

In this study, the pyrolysis properties of PP-wound tapes with a semiconductive buffer layer for cables were investigated by photoionization MS. HZSM-5 catalysts with mass ratios of 20%, 30%, 40%, and 50% were used to conduct pyrolysis experiments at 300, 400, 500, and 600 °C, respectively, and a series of data graphs on the pyrolysis process of PP were obtained. Through our analysis of the mass spectrum, our experiments showed that the combination of PP/40% HZSM-5 has the best pyrolysis efficiency among the four kinds of catalyst ratios. Between the four pyrolysis temperatures, the reaction condition of 400 °C was the most favorable to the pyrolysis reaction of PP. Similarly, we also summarized the mechanism of thermal degradation of PP and better explained its pyrolysis process.

The findings of this study support the notion that the method of photoionization mass spectrometry of pyrolysis products can be used to quickly and accurately analyze the mechanism of polymer pyrolysis and the important factors affecting the pyrolysis process, which provides new research ideas for the buffer layer ablation mechanism and its utilization, such as in the recycling of the polymer in cables.

## Figures and Tables

**Table 1:** Basic information about the polymer and catalyst used in this work.

Name	Manufacturer	Parameter
PP	Shanghai Liyang Machinery and Electric Co. Ltd. (Shanghai, China)	ρ = 0.91 g/cm^3^ Melting point = 161 °C Pore size < 180 μm
HZSM-5	Nankai University Catalyst Co. Ltd. (Tianjin, China)	Si/Al = 36 Surface area = 320 m^2^/g Pore size = 0.55 nm

**Table 2:** Main products of PP pyrolysis.

MW	Formula	Product	Molecular Formula	Volume Fraction at 400 °C (%)
28	C_2_H_4_	ethylene	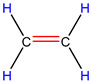	0.09
40	C_3_H_4_	allene	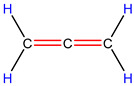	0.08
42	C_3_H_6_	propylene	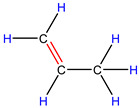	13.44
54	C_4_H_6_	1,3-butadiene	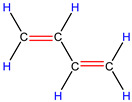	0.14
56	C_4_H_8_	2-methyl-1-propene	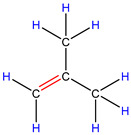	7.19
68	C_5_H_8_	pentadiene	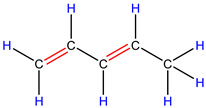	8.26
70	C_5_H_10_	2-pentene	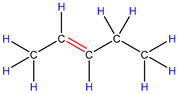	9.22
78	C_6_H_6_	benzene	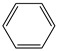	0.27
82	C_6_H_10_	hexadiene	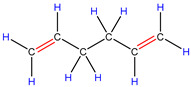	5.30
84	C_6_H_12_	2-methyl-1-pentene 4-methyl-1-pentene	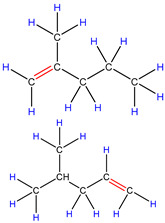	19.95
92	C_7_H_8_	toluene	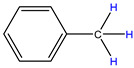	0.26
104	C_8_H_8_	styrene	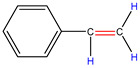	0.06
106	C_8_H_10_	xylene	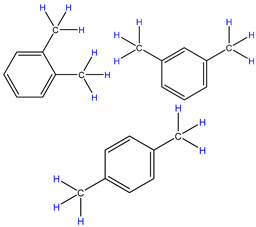	0.13
112	C_8_H_16_	4-methyl-2-heptene	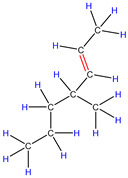	6.20
126	C_9_H_18_	2,4-dimethyl-1-heptene 4,6-dimethyl-2-heptene	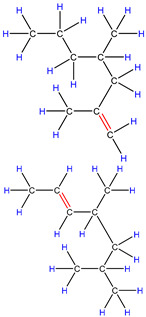	27.63
140	C_10_H_12_	2,4,6-trimethyl-1-heptene	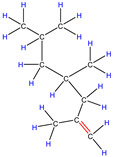	1.76

## Data Availability

Data are contained within the article.
